# Reconstruction of A Type IIIB Hypoplastic Thumb with A Huber Opposition Transfer in A Five-Year-Old Girl: Redefining Surgical Treatment

**DOI:** 10.29252/wjps.8.1.97

**Published:** 2019-01

**Authors:** Joseph A. Ricci, Naman S. Desai

**Affiliations:** 1 Division of Plastic Surgery, Department of Surgery, Brigham and Women’s Hospital, Harvard Medical School, Boston, MA; 2 Department of Radiology, Brigham and Women’s Hospital, Harvard Medical School, Boston, MA

**Keywords:** Congenital hand deformity, Hypoplastic thumb, Huber opposition transfer

## Abstract

Thumb hypoplasia, a congenital deficiency of bony and soft tissue of thumb, is often associated with systemic syndromes like Holt-Oram syndrome, and is the second most common thumb anomaly after duplicated thumb. It has traditionally been classified into six categories, which help guide treatment including reconstruction versus pollicization (transfer of the index finger to thumb). Amputation of the thumb and pollicization is the traditional treatment for a IIIB hypoplastic thumb. A five-year-old girl presented with a classic type IIIB hypoplastic thumb in which she has absent motor function, aplasia of the metacarpal, shortened first web space, and an unstable but present carpometacarpal (CMC) joint. Instead of amputation, the thumb was reconstructed by capsulodesis to stabilize the CMC joint, Huber Transfer Opponensplasty and abductor pollicis longus transfer to restore motor function, W-plasty to deepen the first web space, and distraction to lengthen the metacarpal. The patient tolerated the multi-stage reconstruction and bony distraction well. She was followed for one year postoperatively and has demonstrated a functional thumb. This case questions the surgical algorithm for hypoplastic thumbs and suggests a revised classification system for hypoplastic thumbs which would further divide class III based on not only the stability of the CMC joint but the presence or absence of the joint as well. We propose that amputation be reserved for type III hypoplastic thumbs in which the CMC joint is absent, (revised class IIIC) and reconstruction be attempted when the joint is present irrespective of stability (revised classes IIIA and IIIB).

## INTRODUCTION

Thumb hypoplasia is defined by an underdevelopment of bony and soft tissue in the first ray. After duplicated thumb, hypoplastic thumb is the second most common thumb anomaly with 60% occurring bilaterally.^1^^,^^2^ It may be isolated or secondary to a deficiency of the radial side of the upper limb or to constriction ring syndrome.^3^ Hypoplastic thumbs are associated with systemic syndromes such as Holt-Oram syndrome, Fanconi anemia, and the vertebral, anal, tracheal, esophageal, phalangeal, and renal (VATER) anomalies in 18 to 43 percent of the patients.^4^

This condition has traditionally been classified by Blauth-Buck-Gramcko and Manske into five types(six subgroups)to guide treatment (Table 1).^5^ Type I, the least severe, demonstrate minimal shortening and narrowing of the thumb and often does not require surgical intervention. Type II thumbs are characterized by a hypoplastic thenar musculature, narrowing of the first web space, and an unstable metacarpophalangeal (MCP) joint. Type III thumbs are divided into a subtype IIIA and IIIB. Type IIIA thumbs are similar to Type II with the addition of extrinsic tendon abnormalities, but have a stable carpometacarpal (CMC) joint. This is in contrast to Type IIIB, which have an unstable CMC joint as well as partial metacarpal aplasia. Type IV thumbs, referred to as *pouce flottant* or floating thumbs, have rudimentary bony and soft tissue structures with an intact neurovascular bundle. Type V thumbs are absent thumbs.^6^

Classically, the severity of hypoplasia dictates treatment options. In less severe cases, TypesI, II and IIIA, motor function is strengthened by muscle transfers to improve abduction and opposition and the first web space is deepened to enhance pinching and grasping. In severe cases of hypoplasia (Types IIIB, IV and V), the treatment is amputation of the rudimentary thumb combined with pollicization of the index finger.^1^^-^^6^ Vascularized toe-to-thumb transfer has also been described for Type IIIB thumbs.^7^^-^^9^ Pollicization is used for patients with a congenitally absent or non-functional thumb. The goal of the procedure is to improve the appearance of the hand and the patient’s ability to grasp large objects as children without functional thumbs can manipulate small objects using a side-to-side pinch between the index and long fingers.^10^ Focused on a similar goal but operating contrary to the traditional treatment paradigm, we present a patient with a type IIIB thumb who underwent reconstruction to establish a functional and usable thumb rather than amputation and pollicization.

## CASE REPORT

**Fig. 1:**
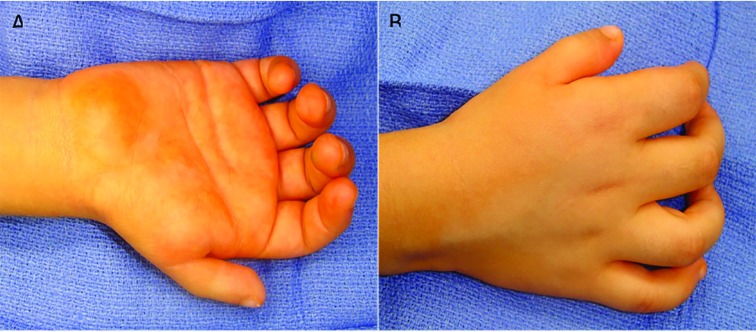
Volar (A) and dorsal (B) preoperative photographic views of a 5-year-old girl with a non-usable right thumb since birth.

**Fig. 2:**
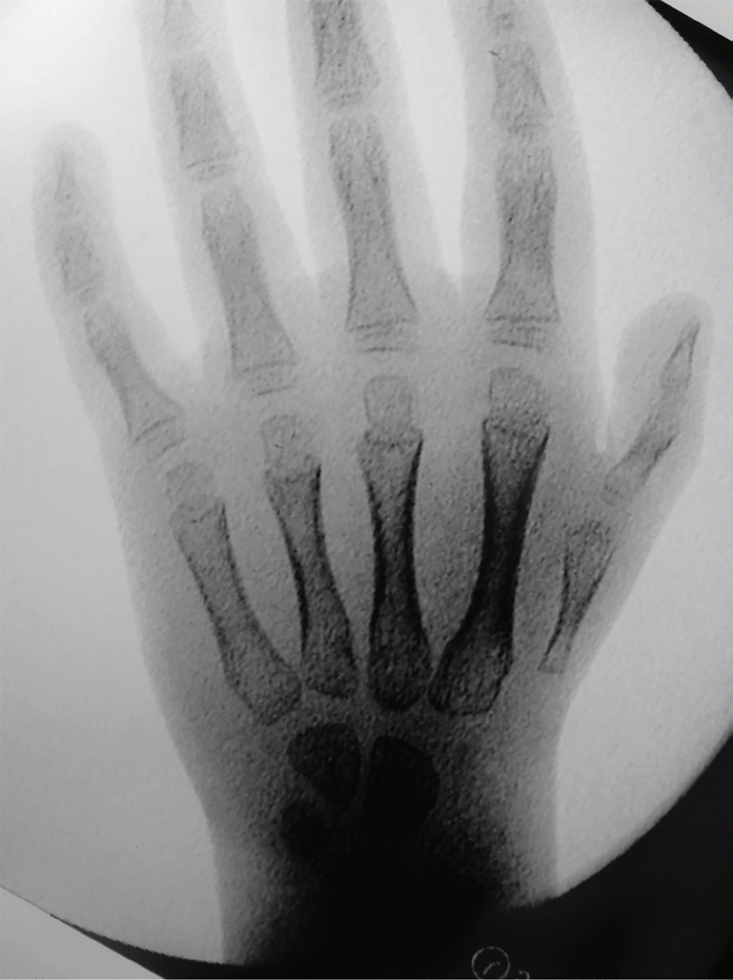
AP intra-operative x-rays demonstrate aplasia of the radial portion of the carpus with a hypoplastic first metacarpal

A 5-year-old girl was referred because she had a non-usable right thumb since birth. The patient had no other congenital anomalies, no family history of anomalies and had no previous surgical intervention. On physical exam, the patient had a small and thin right thumb when compared to the left (Figure 1A and B). The thumb had no motor function (no extensor, flexor or abductor motion) and was markedly unstable due to hypoplasia of capsular ligaments around the CMC joint. Intra-operative x-rays demonstrate aplasia of the radial portion of the carpus with a hypoplastic first metacarpal (Figure 2). By clinical and radiographic examination, the patient had aclassic Type IIIB hypoplastic thumb. Over a 15-month period, the patient underwent a staged surgical treatment to reconstruct the thumb. 

**Fig. 3:**
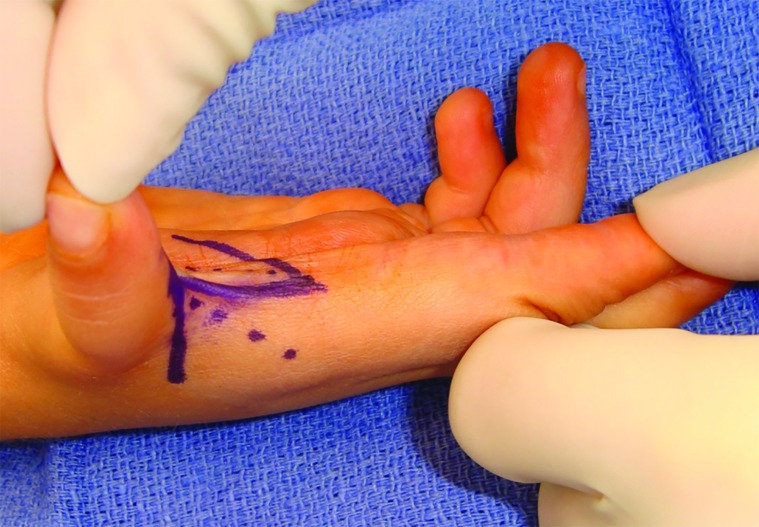
A W-plasty was performed to deepen the web space between the thumb and index finger to enhance thumb abduction, pinching, and grasping

At the initial procedure, a longitudinally placed W-plasty was performed to deepen the web space between the thumb and index finger (Figure 3). Skin flaps were elevated and transposed to create a contoured deepening of the first web space to enhance thumb abduction, pinching, and grasping. Secondary to hypoplastic capsular ligaments, the thumb metacarpal base was markedly unstable, necessitating a capsulodesis procedure. The metacarpophalangeal (MP) joint was exposed and the joint capsule incised. The volar plate was elevated in a T-shaped fashion enabling the two volar plate flaps to be imbricated over one another in a vest over pants fashion, preventing hyperextension. This step repositioned the MP joint from a hyperextended, unstable state to a flexed configuration that enhances tip pinch. Great care to preserve the joint surfaces as well as the epiphyseal plates.

**Figure. 4:**
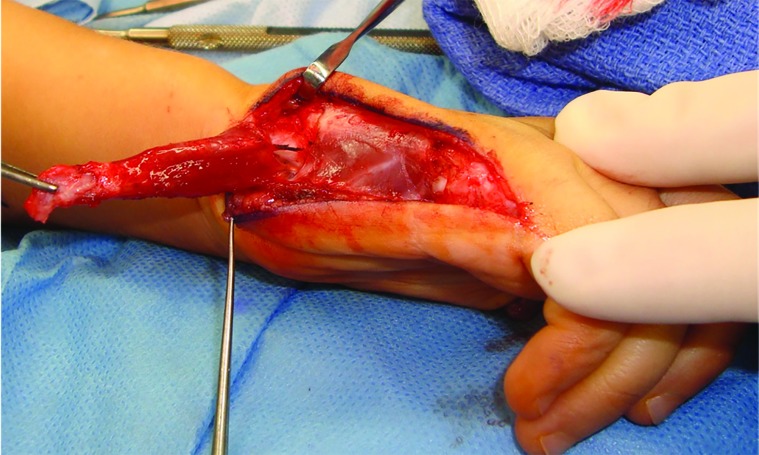
Harvest of abductor digiti minimi by transection of its distal insertion at the base of the fifth proximal phalanx for use in Huber Transfer Oppopensplasty

A Huber Opponensplasty was also performed at the index procedure. The hypothenar muscle flap was harvested utilizing a mid-axial incision along the ulnar border of the fifth metacarpal. The distal extensor insertion of the abductor digiti minimi was transected near its insertion in the base of the fifth proximal phalanx. The extensor mechanism was left intact and preserved, as was the short flexor to the fifth finger. The muscle was elevated, maintaining its pisiform attachment and ulnar neurovascular pedicle. The muscle was then inverted in the same manner as turning the page of a book (Figure 4). A subcutaneous tunnel connecting the thumb incision to the ulnar mid-axial incision was created. The muscle flap was delivered through this subcutaneous pocket in a parallel vector to the shaft of the thumb metacarpal. With the thumb in maximal abduction, the transposed muscle was sutured to the conjoint tendon just proximal to the MP joint. This position will alleviate stress from the hypoplastic collateral ligaments.

Due to the absence of a thumb extensor, the abductor pollicis longus also was transferred to the extensor pollicis longus tendon to recreate a thumb extensor. On the dorsum of the wrist, the interval over Lister’s tubercle was incised. The extensor pollicis longus was elevated. It was noted to be markedly hypoplastic, without proximal motor function or excursion. It was divided proximal to Lister’s tubercle. The anomalous junctura tendineae attaching the thumb extensor pollicis longus to the extensor indicis proprius were divided to prevent multiple actions on the tendon transfer. Next, an incision was made over the first dorsal compartment just proximal to the radial styloid. The retinaculum was incised and the abductor pollicis longus tendon was exposed and elevated. 

The extensor pollicis longus tendon was then transferred from the third dorsal compartment to the first. A transverse incision in the abductor pollicis longus was made to allow for an intra-tendinous weave with the extensor pollicis longus tendon. This was done with the thumb in a palmarly extended position, though the IP distal insertion was deemed not to be entirely mobile and the excursion was not normal. At this step, further dissection was not performed as there had been multiple incisions both palmarly and dorsally about the base of the thumb. Thus, most of the pull or transferred power from the abductor pollicis longus tendon transfer was inserted more through the extensor pollicis brevis insertion at the base of the proximal phalanx as opposed to at the base of the distal phalanx. Following completion of the intra tendinous weave and suture, all wounds were closed, and the extremity was protected with a thumb gauntlet splint.

**Fig. 5:**
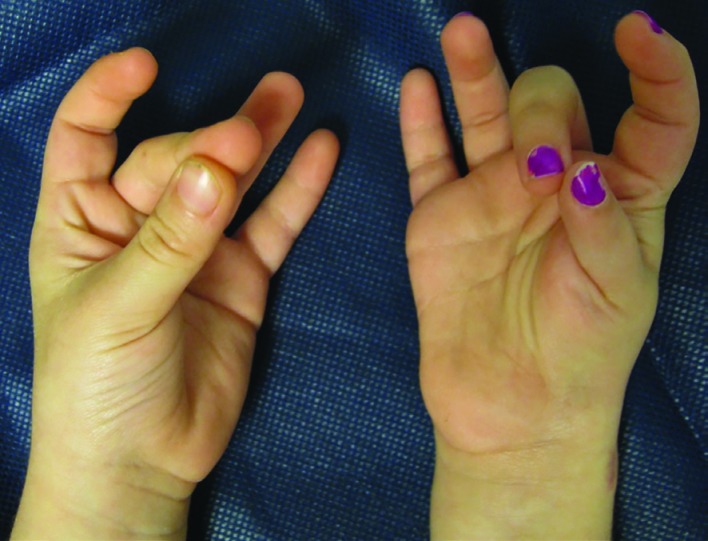
After the first stage of the reconstruction, the patient is able to oppose her thumb of her right hand to the middle finger without difficulty

Fourteen months later, the patient was doing well and returned for the second stage procedure. The previous surgical procedures enabled the child to tip pinch from the thumb to the index and long fingers (Figure 5). Due to the short stature of the thumb, it was difficult to touch her ring and small fingers. The thumb metacarpal was stressed demonstrating instability in the base of the thumb metacarpal. The small thumb prevented the patient from being able to pinch large diameter objects. Additionally, there was aplasia of the radial portion of the carpus creating an unstable carpal-metacarpal joint. The treatment plan was to create an osteotomy of the right thumb metacarpal and apply a uni-planar distraction lengthener to elongate the bone as well as stabilize the thumb carpal-metacarpal joint with longitudinal Kirschner wires.

**Fig. 6:**
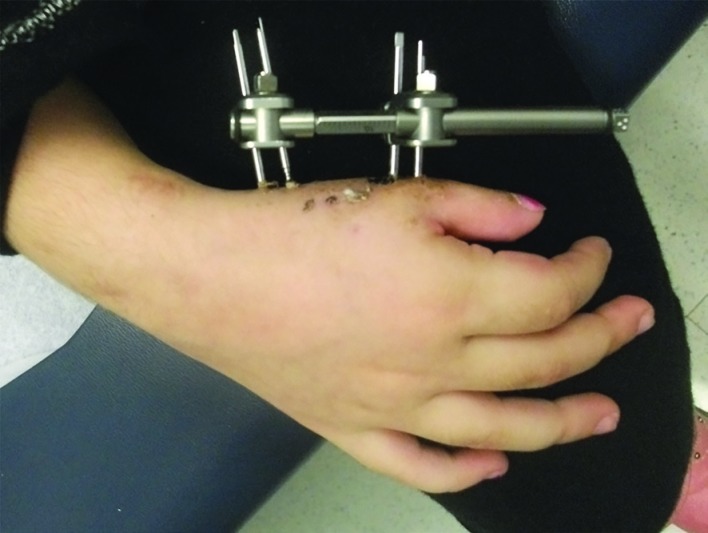
A mini Stryker distraction lengthener for digits was attached to the thumb metacarpal bicortically. The device was turned 1 mm/day for three months to achieve a length of 4 cm

First, the mini Stryker (Kalamazoo, MI) distraction lengthener for digits was placed into its most compressed position (Figure 6). Four pins were placed through stab wounds and drilled into the thumb metacarpal bicortically. The device was mounted onto the half pins and a small incision was then made 90 degrees off axis from the pin placement to expose the metacarpal shaft for the osteotomy. The osteotomy was performed between the pins, being careful to preserve the periosteal blood flow by minimizing periosteal stripping. A 1.2-mm K-wire was used to make multiple drill holes across the metacarpal shaft, that are subsequently connected with an osteotome. 

A K-wire was passed in an antegrade fashion out through the metacarpal canal, the osteotomy reduced, and then the K-wire was passed down the shaft to the metacarpal base so that the base of the thumb would be abducted and aligned with the trapezium and scaphoid. A second wire was inserted into the base to provide two-point fixation, stabilizing the thumb in a tip pinch position during the lengthening process. The lengthener was then expanded to confirm that the osteotomy could distract along the longitudinal wire and then repositioned. Its position was verified with anteroposterior (AP) and lateral views with the image intensifier. Incisions were sutured, and the patient was placed in a thumb spica splint.

The thumb lengthener was turned 1 mm a day. After three months of distraction and healing, the bone was at goal length (an additional 4 cm) and appeared united. The patient returned to the operating room to remove the hardware and buried K-wires. Following removal of hardware, the thumb metacarpal lengthened segment was noted to be stable in all positions, signifying that additional bone grafting was not required. The patient was followed in clinic for 1 year after the procedure. She has learned to use her restored thumb for pinching and grasping. Although weaker than her normal thumb, her right thumb was functional.

## DISCUSSION

Hypoplastic thumb deformities represent of spectrum of anomalies making the surgical treatment sometimes unclear. Classically, surgical reconstruction was proposed for Types II and IIIA hypoplasia, while amputation and pollicization was reserved for Types IIIB and IV. But pollicization may not be the best treatment for all children with Type IIIB deformities. Research has demonstrated that pollicization leads to decreased strength and functional integration.^11^^-^^15^ When compared to a normal thumb, pollicized thumbs only attain 21% strength in grasping and 22-26% in pinching. Seventy-seven percent of patients demonstrate good pulp-to-pulp opposition but thumb mobility is limited to 50% of the normal side.^11^

Thumb reconstruction instead of amputation and pollicization for patients with a revised Type IIIB hypoplastic deformity has not been extensively explored in the literature. Only one study by Foucher questions if reconstruction could be an option. They retrospectively reviewed five cases where patients with a revised Type IIIB thumb underwent a two-stage reconstruction using a free vascularized metatarsophalangeal joint in the first stage followed by classic non-microsurgical steps. In their opinion, reconstruction of a revised type IIIB thumb should be reserved for the patient who requests improvement of a non-excluded thumb or whose family absolutely refuses to accept pollicization.^7^

In opposition to traditional doctrine where a revised Type IIIB hypoplastic thumb should be pollicized, this case report exhibits a two-stage reconstruction of the thumb of a 5-year-old girl without the use of microsurgical technique. In this case, the patient presented because she had a non-usable thumb and her parents refused amputation and pollicization. Given the present although unstable CMC, we aimed to stabilize the joint so that functionality could be restored.

The Manske classification of hypoplastic thumbs is predicated on the presence (Type IIIA) or absence (Type IIIB) of CMC joint stability.^5^

This classification has led to the treatment algorithm of restoration versus pollicization. Based on the successful results of this patient, we wish to challenge this classification, redefining surgical options for children born with a Type III deformity. We propose that Type III deformities should be divided into three categories: stable CMC joint, unstable but present CMC joint, absent CMC joint (Table 2). As demonstrated in this case report, the presence of the CMC joint allows for restoration of stability through capsulodesis. Once stable, the thumb can be powered by muscle transfers to recreate a functional digit. A revised Type IIIB thumb presents a subset of patients in whom reconstruction can be achieved. Future studies would have to compare patients with Type IIIB anomalies who underwent pollicization versus restoration. This new outlook on surgical options may lead to improving surgical outcomes regarding strength, function, and cosmesis.

## References

[1] James MA, McCarroll HR Jr, Manske PR (1996). Characteristics of patients with hypoplastic thumbs. J Hand Surg Am.

[2] Lister G (1991). Pollex abductus in hypoplasia and duplication of the thumb. J Hand Surg Am.

[3] Tay SC, Moran SL, Shin AY, Cooney WP 3rd (2006). The hypoplastic thumb. J Am Acad Orthop Surg.

[4] Abdel-Ghani H, Amro S (2004). Characteristics of patients with hypoplastic thumb: a prospective study of 51 patients with the results of surgical treatment. J Pediatr Orthop B.

[5] Manske PR, McCarroll HR Jr, James M (1995). Type III-A hypoplastic thumb. J Hand Surg Am.

[6] Graham TJ, Louis DS (1998). A comprehensive approach to surgical management of the type IIIA hypoplastic thumb. J Hand Surg Am.

[7] Foucher G (1999). Prospects for hand transplantation. Lancet.

[8] Nishijima N, Matsumoto T, Yamamuro T (1995). Two-stage reconstruction for the hypoplastic thumb. J Hand Surg Am.

[9] Shibata M, Yoshizu T, Seki T, Goto M, Saito H, Tajima T (1998). Reconstruction of a congenital hypoplastic thumb with use of a free vascularized metatarsophalangeal joint. J Bone Joint Surg.

[10] Manske PR (2010). Index pollicization for thumb deficiency. Tech Hand Up Extrem Surg.

[11] Manske PR, McCaroll HR Jr (1985). Index finger pollicization for a congenitally absent or nonfunctioning thumb. J Hand Surg Am.

[12] Buck-Gramcko D (1991). Complications and bad results in pollicization of the index finger (in congenital cases). Ann Chir Main Memb Super.

[13] Clark DI, Chell J, Davis TR (1998). Pollicisation of the index finger A 27-year follow-up study. J Bone Joint Surg Br.

[14] Kozin SH, Weiss AA, Webber JB, Betz RR, Clancy M, Steel HH (1992). Index finger pollicization for congenital aplasia or hypoplasia of the thumb. J Hand Surg Am.

[15] Sykes PJ, Chandraprakasam T, Percival NJ (1991). Pollicization of the index finger in congenital anomalies A retrospective analysis. J Hand Surg.

